# A Neonate with CLOVES Syndrome

**DOI:** 10.1155/2014/845074

**Published:** 2014-10-22

**Authors:** Dilek Sarici, Mustafa Ali Akin, Selim Kurtoglu, Filiz Tubas, Serdar Umit Sarici

**Affiliations:** ^1^Division of Neonatology, Department of Pediatrics, Erciyes University Faculty of Medicine, 38039 Kayseri, Turkey; ^2^Department of Pediatrics, Erciyes University Faculty of Medicine, 38039 Kayseri, Turkey; ^3^Division of Neonatology, Department of Pediatrics, Ufuk University Faculty of Medicine, 06500 Ankara, Turkey

## Abstract

Congenital lipomatous overgrowth, vascular malformations, and epidermal nevi (CLOVE) syndrome is a recently delineated disorder that comprises vascular malformations (typically truncal), dysregulated adipose tissue, scoliosis, enlarged bony structures (typically of the legs) without progression, or distorting bony overgrowth. The name CLOVE was subsequently extended to CLOVES to emphasize the association with scoliosis/skeletal and spinal anomalies and seizures/central nervous system malformations. We herein report a very rare case of CLOVES syndrome with the findings of lipomatous overgrowth in the cheek (facial asymmetry), vascular malformation (hemangiomas), epidermal nevi (large port wine stains), and skeletal abnormalities (widened first interdigital space, dystrophia in the nail of the first digit of the right foot, and bilateral hypertrophy of the first digits of the feet).

## 1. Introduction

Congenital lipomatous overgrowth, vascular malformations, and epidermal nevi (CLOVE) syndrome is a recently delineated disorder that comprises vascular malformations (typically truncal), dysregulated adipose tissue, scoliosis, enlarged bony structures (typically of the legs) without progression, or distorting bony overgrowth [1]. The name CLOVE was subsequently extended to CLOVES to emphasize the association with scoliosis/skeletal and spinal anomalies and seizures/central nervous system malformations [2]. We herein report a very rare and most recently defined case of CLOVES syndrome.

## 2. Case Report

**Figure 1:**
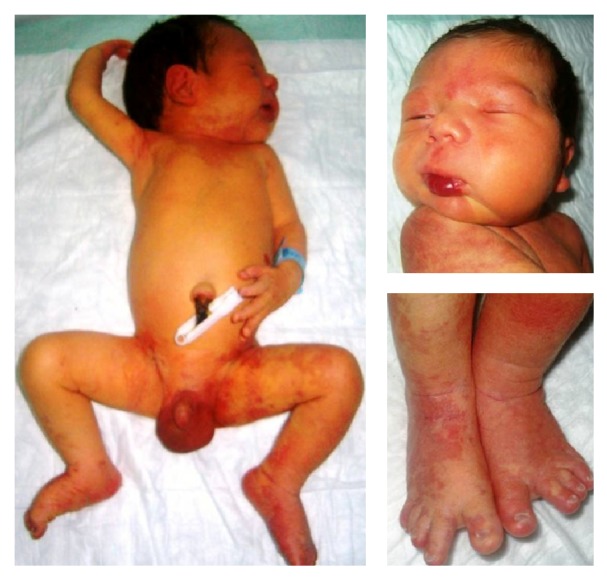
Common port wine stain partially involving the skin overlying the right arm, sternal region in the neck, right temporooccipital region, right leg, anterior and posterior parts of left leg, an hemangioma with a size of 2 × 1 cm in his lower lip, hypertrophy on left cheek, widened first interdigital space, dystrophia in the nail of the first digit of the right foot, and bilateral hypertrophy of the first digits of the feet.

A male newborn who had been diagnosed to have hydrothorax on fetal ultrasonography was born to a 29-year-old woman at 39 weeks' gestation with a birth weight of 3400 g and transferred to our neonatal intensive care unit as he had common port wine nevus on his trunk. Antenatal history of the mother was uneventful except for smoking 1-2 cigarettes per day, and she had upper airway and urinary tract infections one week before the birth. There was no consanguinity between the parents. On physical examination he had a common port wine stain partially involving the skin overlying the right arm, sternal region in the neck, right temporooccipital region, right leg, anterior and posterior parts of left leg, and sacral region. Additionally he had a systolic murmur with a grade of II/VI, an hemangioma with a size of 2 × 1 cm in his lower lip, hypertrophy on left cheek, widened first interdigital space, dystrophia in the nail of the first digit of the right foot, and bilateral hypertrophy of the first digits of the feet (Figure 1). Ultrasonography (USG) of the left cheek revealed lipomatosis. Results of the abdominal and transcranial USGs and cranial magnetic resonance imaging (MRI) were unremarkable. Echocardiography demonstrated patent foramen ovale and asymmetric septal hypertrophy. Thoracic USG detected right pleural effusion. However the patient had no respiratory distress and the effusion resolved spontaneously on follow-up. The diagnosis of CLOVES syndrome was established on the basis of the findings of lipomatous overgrowth in the cheek (facial asymmetry), vascular malformation (hemangiomas), epidermal nevi (large port wine stains), and skeletal abnormalities (widened first interdigital space, dystrophia in the nail of the first digit of the right foot, and bilateral hypertrophy of the first digits of the feet) and the patient was put on an outpatient follow-up program.

On a control visit at 3 months of age a 2 cm increase (lipomatous mass) was detected in the circumference of left leg in comparison to right leg.

## 3. Discussion

The pathogenesis of CLOVES has just been identified. Kurek et al. [3] identified mutations in* PIK3CA* in six patients with CLOVES syndrome, and mutant allele frequencies ranged from 3% to 30% in affected tissue from multiple embryonic lineages. They conclude that CLOVES is caused by postzygotic activating mutations in PIK3CA [3]. Further studies are needed to support these mutations. We could not study this mutation in our patient as the presence of this mutation was published after the diagnosis of the present case and the patient was lost on long-term follow-up.

In the cohort reported by Alomari [4], amongst 18 patients with CLOVES syndrome, the most prominent features were truncal lipomatous masses of variable size, complex and potentially debilitating vascular malformations (including lymphatic, arteriovenous, and phlebectasia), scoliosis, and skeletal and other anomalies. In addition to the vascular anomalies, there is a wide spectrum of imaging findings in CLOVES syndrome reflecting the asymmetric body overgrowth and musculoskeletal and other internal organ anomalies. Musculoskeletal findings include extremity bony and soft tissue overgrowth, leg length discrepancy, chondromalacia patellae, dislocated knees, scoliosis, wide triangular feet with widened first interdigital space or large hands, and macrodactyly, typically involving the third toe or third finger, talipes, and neural tube defect. Renal hypoplasia is also frequently seen [4]. Our patient had wide triangular feet with widened first interdigital space, dystrophia in the nail of the first digit of the right foot, bilateral hypertrophy of the first digits of the feet, and hypertrophy on left cheek.

The lipomatous masses, characteristic of the CLOVES syndrome, behave more like tumors and, thus, are inclined to enlarge and recur after resection [4]. Our patient had lipomatous mass on his face. A port wine stain partially involving the skin overlying the right arm, sternal region in the neck, right temporooccipital region, right leg, and anterior and posterior parts of left leg and a wide port wine stain with irregular borders in sacral region were noted in this patient.

Management in CLOVES syndrome is mainly supportive. A multidisciplinary collaboration of plastic surgeon, dermatologist, and pediatrician is required. Asymmetrical lower limb growth may lead to kyphosis and scoliosis. The increased risk of pulmonary embolism has been described in patients with CLOVES syndrome [5]. Long-term prognosis is still unclear, and much more data and experience are necessary in this respect.

We herein report this case as these cases are extremely rare. We recommend that if a baby has a port vein stain, overgrowth in some parts of the body, hemangioma in the lip, and foot deformities, CLOVES syndrome should be considered in differential diagnosis.
